# Associations Between Built Environment Characteristics and Walking in Older Adults in a High-Density City: A Study From a Chinese Megacity

**DOI:** 10.3389/fpubh.2020.577140

**Published:** 2020-11-05

**Authors:** Hui He, Tingting Li, Yanwei Yu, Xiaowu Lin

**Affiliations:** ^1^School of Architecture and Urban Planning, Huazhong University of Science and Technology, Wuhan, China; ^2^Xiamen Urban Planning & Design Institute, Xiamen, China

**Keywords:** high-density city, walking, transport-related walking, leisure-time walking, older adults, walkability

## Abstract

The built environment is an important factor affecting physical activity, especially walkability. Walkability is used to characterize the user friendliness of outdoor physical activity. However, studies on walkability and physical activity are mainly concentrated on low-density Western cities. Study on the walkability of high-density cities in Asia, especially with the elderly, is seriously lacking. And walkability is often used as a composite indicator. This study mainly re-examines the relationship between the common indicators of walkability (population density, street connectivity, land-use mix, and retail density), transport-related walking, and leisure-time walking with older adults in China's megacities. Twelve housing estates in Wuhan were selected for study areas. We explored the association between the walking activities of 1,161 elderly people (≥60 years old) and the indicators of walkability in their neighborhoods. Socio-demographic characteristics were controlled in the multilevel logistic regression models of the built environment walking associations. We found that there was no significant correlation between the four indicators of walkability and transport-related walking. Street connectivity is significantly positively correlated with the participants' leisure-time walking (OR = 1.499, 95% CI = 1.068~2.103), and there was no significant correlation between the other indicator of walkability and leisure-time walking. The results show that there was no statistical correlation between walkability and transport-related walking in the elderly, and only one indicator was related to leisure-time walking. It is extremely important to re-examine the characteristics of built environments and elderly walking activities in high-density cities. Only by implementing effective intervention strategies in different urban backgrounds can cities move toward a more active and healthier path.

## Introduction

At present, the acceleration of population aging has become a common phenomenon in megacities around the world. How to deal with the negative impact of population aging is a serious challenge facing humanity. According to a report released by the World Health Organization (WHO), the elderly population will exceed that of children, and 80% of the elderly will live in developing countries by 2050 (1). Chinese population aging is much faster than many middle and low income countries, and China's population aging has two characteristics: on the one hand, the process is accelerating in recent years; on the other hand, the number will triple (402 million people) by 2040 (2). Under the current situation of global aging and the shortage of medical resources, encouraging older people to participate in physical activities to improve their health is of greater significance than clinical treatment. The World Health Organization encourages the elderly to participate in at least 150 min of aerobic physical activity every week to protect their health (3). Empirical studies have shown that regular and adequate levels of physical activity can provide mental and physical health benefits and can also reduce the risk of many chronic diseases (4, 5). Despite such obvious benefits of physical activity, a WHO global health survey found that the percentage of the population that meets the recommended amount of exercise is decreasing with age (6). In 2013, nearly 50% (100 million) of older people in China experienced non-communicable diseases. According to the 2010 Chronic Disease Risk Factor Surveillance Survey, nearly 84% of older people do not engage in regular physical activities. And there was a marked difference among older people in urban (24%) compared to rural areas (7.1%) (7). The combination of population aging and physical inactivity reduces the quality of life of older adults and increases the burden of health care. Therefore, from the perspective of public health, it is essential to explore the relevant factors that promote the physical activity of the elderly and propose effective intervention strategies.

Compared with people of other ages, the elderly are often physically impaired and generally do not commute. They often engage in outdoor activities in spaces near their residential area (8, 9), so the characteristics of the neighborhood environment can affect the physical activity of the elderly. It is essential to explore the relationship between built environment and physical activity of the elderly in order to guide the construction of age-friendly communities to promote physical activity of the elderly, which will help maintain good physical function of the elderly and thus prolong independent community life. In the theoretical study of social ecological models, the built environment is one of the most important factors affecting physical activity (10, 11). In a number of systematic reviews, the researchers determined that built environment characteristics are related to physical activity of the elderly, including walkability, overall access to destinations and services, land-use combinations, and a walk-friendly infrastructure (12, 13). Most research cites two definitions of measurement range of a neighborhood environment. One defines the buffer zone geographically (generally within a 1,000 m buffer zone around the participant's residence). A study from Hong Kong defined an 800 m circular buffer zone of the neighborhood (14), and a study from Seattle defined a 500 m circular buffer zone (15). Researchers generally use GIS tools to audit the environmental attributes of a buffer zone. The second way of defining a measurement range is from the home starting point of participants to a self-aware walking area of 10 to 20 min (16, 17). The general neighborhood environmental perception questionnaire is used to assess the environmental attributes.

The 3D (Density, Diversity, and Design) environmental elements have also been shown to relate to the physical activity of the elderly (especially walking activities) in land-use mix, population density, street connectivity, and retail facilities (18–20). In the study of low-density cities in western developed countries, it was found that the above environmental factors are more positively related to the promotion of outdoor physical activities. Therefore, based on research results of the 3D elements, the researchers put forward a composite indicator of walkability that characterizes the degree of friendliness of the built environment to the physical activities of residents (21, 22). The combined indicators of walkability generally include residential density, street connectivity, land-use mix, and the retail building area ratio (23, 24). When calculating walking ability, the measured values are standardized and then weighted together (25, 26).

As mentioned earlier, relevant research between the indicators of walkability and physical activity is carried out frequently on low-density cities in the West, and most research results show that indicators of walkability promote residents' physical exercise. However, there are relatively few studies on high-density cities in China, especially China's megacities. What is more, the population density of China's megacities is much higher than that of many cities in Western countries, and the differences between urban built environments and culture may result in different research results. Taking population density as an example, academic circles have found relevant research results in China's megacities: some research results show that it is negatively correlated with adolescents' entertainment physical activity (27, 28), or they show no correlation (29); another study found that it is negatively related to the leisure physical activities of adult women (30). Therefore, it is particularly important to re-examine the relationship between the built environment of megacities and the physical activity of residents. Research should focus on the elderly because the population aging degree in China's megacities is acute. It is critical to intervene in the health of the elderly from the aspect of built environment. The local government should promote the physical activity of the elderly from the aspects of planning policy and urban design, which can reduce the pressure of elderly care (31).

Related research shows that walking is the most popular physical activity for the elderly (32), so this study focuses on the relationship between elderly walking activities and the built environment. Two points need to be emphasized. First, the existing calculation formula of walkability is based on the fact that all indicators are positively correlated with physical activity, but some indicators of walkability may not be positively correlated with physical activity. Secondly, current research mainly studies the built environment from the perspective of a single type of physical activity, and there is a lack of comparison between different categories. In this study, however, the elderly walking activities are divided into transport-related walking and leisure-time walking, and we compare the differences of the relationship between built environment and two types of walking activities in the elderly.

In summary, this study examined the relationship between two types of walking and the walkability index of the elderly in 12 residential areas in Wuhan, China. We hypothesize that there are positive relationships between the four indicators of walkability and both types of walking based on the findings of previous studies.

## Methods

### Study Areas and Sampling Approach

**Figure 1:**
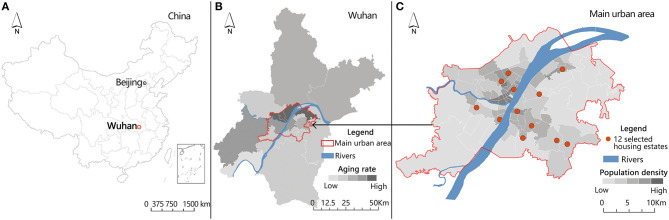
**(A)** Location of Wuhan in China; **(B)** Proportion of the aging population in different administrative districts of Wuhan; and **(C)** The 12 selected housing estates in Wuhan.

Wuhan is a megacity in central China (Figure 1A). The proportion of the aging population of Wuhan has been increasing in the past two decades, and the aging rate of 7 administrative districts located in the main urban area has exceeded 20% since 2018. The main urban area of Wuhan has a much higher aging rate than the suburban areas (Figure 1B) (33). The population density of the main urban area of Wuhan is 5,898~25,790 people/km^2^ (34), the minimum population density (5,898 people/km^2^) in our study is still much higher than 500 persons/km^2^, the cutoff for high residential density in Western countries (35). During the selection, we chose residential areas with a high aging rate so that the researchers could collect enough data. We also sought to select housing estates with similar homogeneous socioeconomic (SES) profiles; thus, we selected housing estates with a similar median house price. Based on those criteria, we selected 12 housing estates in the main urban area as our sample (Figure 1C).

We conducted the study from October to November 2019, when the weather is cool and pleasant for walking activities with older adults. Trained interviewers visited the selected housing estates and interviewed 80–120 older adults, using a random sampling method in each housing estate. All participants were able to engage in physical activity independently and had lived in the residence for over 1 year. A total of 1,161 valid questionnaires were collected.

### Walking Data

The times the adults walked were obtained through questionnaire interviews. The content and form of the questionnaire were optimized based on the International Physical Activity Questionnaire. Because older adults may have difficulty reading or filling out the questionnaire, the survey was completed by trained interviewers after face-to-face interviews with participants. The survey mainly investigated the transport-related walking activities and leisure-time walking activities of the elderly in the neighborhood. The following four questions were asked:

1. In the last 7 days, how many days have you carried out transport-related walking for at least 10 min (e.g., walking to the bus station, shopping, seeing a doctor, etc.)?
2. How much time do you usually spend walking in a day when you have transport-related walking?
3. In the last 7 days, how many days did you have a leisure-time walking activity that lasted for at least 10 min (excluding the transport-related walking activities mentioned in question 2)?
4. How much time do you usually spend a day on leisure-time walking?

We multiplied the average duration of walking time (in minutes) by the number of days engaged in walking in the past 7 days to obtain the total time of the two types of walking in older adults. As the total duration distribution of the two types of walking activities was highly skewed, with many participants reporting few transport-related walking (38.16%), but much leisure-time walking (66.93%) being reported, the total minutes of walking time per week was transformed into binary variables. We transformed transport-related walking into binary categorical variables of ≥150 vs. <150 min/per week [WHO recommends that the elderly exercise “150 min/week” to protect their health (3)], and we transformed leisure-time walking into binary categorical variables of ≥150 vs. <150 min/per week.

### Built Environment Variables

The built environment variables select common indicators in walkability, including population density, street connectivity, land-use mix, and retail density. The measurement range of environment variables is within an 800 m circular buffer zone around the housing estate of participants. The selection of the 800 m buffer was based on average walking distance and it is the area within 10 to 15 min elderly walking distance. Population density is defined as the resident population per unit of land area where the participant is located. Street connectivity measures the inter-connectedness of the street network within a participant's walkable service area. The measure is a ratio of the count of three (or more) way intersections over the area (km^2^). In the mixed land-use calculation, the land types are mainly divided into three categories: residential, commercial, and office (21); Retail business density measures the convenience of daily shopping within a participant's walkable service area. The four built environment variables are described along with applicable data sources in Table 1.

**Table 1:** Built environment measures.

**Measure**	**Definition**	**Scale of measurement for target area selection**	**Equation**	**Data source(s)**
Population density	Resident population per unit of land area	Administrative Street^a^	Count of resident population/area of administrative Street	The Sixth National Census in Wuhan (2010)
Street connectivity	Number of street intersections per unit of land area	800 m circular buffer	Number of intersections/area of 800 m circular buffer	Baidu Maps (accessed September 17, 2019)
Land-use mix	Evenness of distribution of residential, commercial, and office per unit of land area	800 m circular buffer	Equation below^b^	Urban Master Planning of Wuhan (2010–2020)
Retail density	Number of retail shops per unit of land area	800 m circular buffer	Number of retail shops/area of 800 m circular buffer	Amap (accessed September 10, 2019)

^a^ Administrative street is the smallest unit of urban population statistics in China.

*^b^*
$$\begin{array}{l} land-use\;mix=\frac{(-1)\;\;\times \;\left[\left(\frac{b1}{a})\times \text{ln}(\frac{b1}{a})+(\frac{b2}{a})\times \text{ln}(\frac{b2}{a})+(\frac{b3}{a})\times \text{ln}(\frac{b3}{a}\right)\right]}{\ln\;\;3}. \end{array}$$

a (total square footage of commercial, residential, and office),

b *(square footage of commercial, residential, or office). The formula for land use mix presented ranges from 0 to 1, and a high score indicates high heterogeneity of land use*.

### Individual Covariances

Individual covariates include gender, age, and education level; the participants' ages were transformed into a categorical variable with three levels: 60–69 years (reference category), 70–79 years, and ≥80 years. Education levels were transformed into a categorical variable with four levels: primary school and below (reference category), middle school, high school, and postsecondary school.

### Data Analysis

In this study, 1,210 eligible participants were recruited, while 1,161 completed the survey (response rate = 96%). Multilevel logistic regression models were conducted to investigate the relationship between the built environment and the two types of walking activities for older adults. The house estates were assigned a random effect that accounts for the clustering in the physical activity of participants in the house estates. Model 1 included built environment variables, and Model 2 included further controlled individual covariates. All model analysis results reported Odds Ratios (ORs) and their 95% confidence intervals (95% CI) and *p*-values. These analyses were conducted with R and a multilevel package lme4 (36).

## Results

The descriptive data are shown in Table 2. Approximately 38% of the participants performed at least 150 min of transport-related walking in a week; ~67% of the participants performed at least 150 min of leisure-time walking in a week.

**Table 2:** Descriptive information for participants' walking data, socio-demographic characteristics, and built environment variables.

**Variables**	**Mean (SD)/%**
Outcome (*N* = 1,161)	
Transport-related walking, % ≥150 min	38.16%
Leisure-time walking, % ≥150 min	66.93%
Socio-demographic variables (*N* = 1,161)	
Age	71.22
Gender, % male	46.43%
Education level	
Primary school and below	36.86%
Middle school	26.79%
High school	15.76%
Postsecondary school	20.59%
Built environment factors (*N* = 12)	
Population density (person/km^2^)	25539.00 (10868.01)
Street intersection density (#/km^2^)	15.00 (10.69)
Land-use mix	0.60 (0.16)
Number of retail shops(#/km^2^)	423.00 (233.81)

The results of the multilevel logistic regression model of the participants' transport-related walking time and built environment variables are shown in Table 3. The four indicators of walkability (population density, street connectivity, land-use mix, and retail density) were not significantly correlated with the likelihood of engaging in at least 150 min of transport-related walking in Models 1 & 2. Among the individual covariates, age was significantly related to the likelihood of participating in transport-related walking for at least 150 min. Participants over 70 years old were less likely to conduct transport-related walking than participants who were 60–69 years old (70–79 years old: OR = 0.561, 95% CI = 0.423~0.745; ≥80 years old: OR = 0.242, 95% CI = 0.159~0.368), and middle school education level was negatively associated with the likelihood of participating in transport-related walking for at least 150 min (OR = 0.684, 95% CI = 0.490~0.954). Gender and other education levels had no significant association with the amount of transport-related walking. The direction and magnitude of the effect of built environment variables on transport-related walking time was similar across Model 1 and Model 2.

**Table 3:** Logistic regression of built environment and achieving ≥150 min of transport-related walking a week.

**Model predictor**	**Model 1**			**Model 2**		
	**OR**	**95% CI**	***p*-value**	**OR**	**95% CI**	***p*-value**
**Built environment**						
Population density	0.728	0.508~1.043	0.083	0.788	0.556~1.117	0.181
Street intersection density	1.000	0.725~1.379	0.999	1.091	0.798~1.491	0.586
Land-use mix	1.164	0.850~1.594	0.345	1.164	0.858~1.579	0.330
Number of retail shops	1.183	0.809~1.730	0.386	1.179	0.815~1.706	0.381
**Individual characteristics**						
**Gender**						
Male (reference group)						
Female				1.278	0.986~1.656	0.064
**Age (years)**						
60–69 (reference group)						
70–79				0.561	0.423~0.745	0.000^***^
≥80				0.242	0.159~0.368	0.000^***^
**Education level**						
Primary school and below (reference group)						
Middle school				0.684	0.490~0.954	0.025^*^
High school				1.112	0.756~1.635	0.590
Postsecondary school				0.713	0.497~1.023	0.066
**−2 Log-likelihood**	1524.395			1430.428		

* < 0.05;

** < 0.01;

*** *< 0.001*.

The results of the multilevel logistic regression model of the participants' leisure-time walking time and built environment variables are shown in Table 4. Street connectivity was positively correlated (OR = 1.516, 95% CI = 1.083~2.123 in Model 1; OR = 1.499, 95% CI = 1.068~2.103 in Model 2) with the likelihood of participating in a minimum of 150 min of leisure-time walking, and participants exposed to a high street connectivity were significantly more likely to perform regular leisure-time walking. There was no significant correlation between the other three indicators (population density, land-use mix, and retail density) and the likelihood of participating in a minimum of 150 min of leisure-time walking. Among the individual covariates, postsecondary school education level was negatively associated with the likelihood of participating in leisure-time walking for at least 150 min (OR = 0.649, 95% CI = 0.453~0.929). Gender, age, and other education levels were not significantly related to the possibility of participating in leisure-time walking for at least 150 min a week. The direction and magnitude of the effect of built environment variables on leisure-time walking time was similar across both models.

**Table 4:** Logistic regression of built environment and achieving ≥150 min of leisure-time walking a week.

**Model predictor**	**Model 1**			**Model 2**		
	**OR**	**95% CI**	***p*-value**	**OR**	**95% CI**	***p*-value**
**Built environment**						
Population density	0.777	0.540~1.116	0.172	0.782	0.542~1.127	0.187
Street intersection density	1.516	1.083~2.123	0.015^*^	1.499	1.068~2.103	0.019^*^
Land-use mix	1.325	0.967~1.816	0.080	1.336	0.973~1.835	0.073
Number of retail shops	0.774	0.526~1.139	0.194	0.787	0.533~1.161	0.227
**Individual characteristics**						
**Gender**						
Male (reference group)						
Female				0.875	0.671~1.143	0.327
**Age (years)**						
60–69 (reference group)						
70–79				1.019	0.758~1.370	0.901
≥80				0.953	0.651~1.393	0.802
**Education level**						
Primary school and below (reference group)						
Middle school				0.848	0.603~1.193	0.334
High school				0.974	0.647~1.466	0.900
Postsecondary school				0.649	0.453~0.929	0.018^*^
**−2 Log-likelihood**	1417.000			1386.878		

* <0.05;

** <0.01;

*** <0.001.

## Discussion

### Major Findings

This study further promotes the development of the content of healthy physical activity from two aspects. First, previous research has mainly focused on cities with low- and medium-density populations in the West. This study for a relatively large sample size focused on Wuhan, a megalopolis with a high-density population that has not been previously studied. Second, this study specifically subdivides elderly walking activities into transport-related walking and leisure-time walking so that the different effects of walkability indicators on the different types of walking activities can be showed. The results of this study show that the associations of walkability factors and two types of walking activities are weaker or insignificant in high-density city.

We found leisure-time walking time only related to street connectivity. In this study, street connectivity is positively related to elderly leisure-time walking. Previous studies have shown that streets are the main public space for residents' leisure activities (37, 38). More street intersections provide the elderly with more path options for leisure-time walking, and the elderly choose streets with better space quality for walking activities. There is no significant correlation between population density, land use combination, retail business density and leisure-time walking among the elderly. Some studies have reported the correlation between walkability indicators and leisure-time walking among adults (39). The reason for the differences in the results may be that older adults have relatively more discretionary time to make better use of the relatively good environment around them compared with young people. There may also be another reason that these factors may affect the frequency of walking rather than the time of walking (40).

In our results, we found no significant correlation between transport-related walking time and the four indicators of walkability. According to informal Interviews, old adults have some negative comments on environmental attributes, with respondents citing reasons for not going out, such as too many road cars and speed on the road too fast. These traffic problems may be due to the over-dwelling population leading to increased motor vehicle use in the neighborhood (41). However, the result of our study shows that there is no significant correlation between transport-related walking time and the four indicators of walkability in the elderly, which is similar to the results of a recent study in China (42). The reason may be that for the elderly in Chinese cities, these venues often play an important role in housekeeping during their later years of life, helping families to shop, transport grandchildren to and from school, and accompany grandchildren to outdoor leisure and entertainment activities. Their travel purpose is clear, and these activities belong to the necessary activities of a family, even the old adults are not satisfied with the environment, they also need to travel on a regular basis. This also explains why transport-related walking is related to age. Older people are less likely to undertake outdoor family activities, so older people have less transport-related walking.

Different from the western studies that showed a positive correlation between the indicators of walkability and physical activity, in this study, we found largely non-significant correlations between older adults' walking behaviors and objectively measured built environment factors in walkability. Recent studies conducted in other high-density cities also find these factors tend to be insignificant (14). Therefore, further studies are needed to examine the relationship between factors of walkability and walking for older adults in cities with different urban density. At the same time, some studies in China have shown that population density was negatively correlated with the two types of walking activities (41), but the existing calculation formula of walkability is based on the fact that all indicators are positively correlated with physical activity (43, 44) therefore, the formula may need further study to improve the applicability of walkability.

In addition to the factors we studied, air pollution, traffic noise, traffic safety and crime may also affect older people's walking. There have been a lot of studies on these factors in Western countries (12), but there are still few in China. The built environment and social conditions among countries are greatly different. It is also of great value to study the correlation between these factors and walking among the elderly in China's cities and villages. China's air quality has improved so much since the eco-civilization strategy was introduced. Most people would hardly notice small changes in air quality. But older people with respiratory problems may be more likely to notice. In China, the relationship between traffic noise and walking in the elderly also was rarely studied. For China, such a high population density, crowded traffic countries, it is particularly important to figure out the associations between traffic noise, traffic congestion and elderly walking in order to better provide information for the construction of elderly friendly communities. In China, traffic safety always be mentioned, but there was little mention of crime, which may have something to do with the fact that there are so many people on the streets in China, and there is more surveillance to keep pedestrians safe. However, the large number of vehicles and fast speed were a big safety hazard.

A number of existing studies relied mainly on objective measurements, but there is no consensus on what defines a “neighborhood” (e.g., shape or size). Some studies have reported that the scale and shape of buffers can have an impact on study results (45). However, the use of different buffer radii did not alter the observed relationship in the two studies (46, 47). At the same time, some studies have begun to explore the impact of neighborhood buffer size across various adult life stages (48). However, little attention is focused on the buffer zones of these two types of walking may also differ.

### Limitations

This study has several limitations. First, the cross-sectional research design cannot explain the causal relationship between the high-density urban built environment and elderly walking activities. Second, all measured built environment variables were collected using a single buffer size, and there was a lack of comparison of multiple buffer measurement results. The observed correlation may vary depending on the buffer size, and the range of participants' walking activities may exceed the buffer range. Thirdly, although this study included data on 1,161 individuals, it included only 12 housing estates. This might have affected the power for finding statistically significant associations between environmental attributes and two types of walking. To avoid such a problem, it may be necessary to select more housing estates with each housing estate still including a reasonable number of individuals (49). Finally, the study was conducted in a high-density city in China; and in order to verify the reliability of the results, more evidence from other high-density cities is needed.

## Conclusion

This study examines the relationship between the characteristics of the built environment of high-density cities and the walking activities of the elderly in Wuhan. After emphasizing the different built environmental characteristics and the background of cultural life, it is obvious that the composite indicator of walkability needs to be revised to increase its applicability. Furthermore, the evidence provided by this study will help to clarify the various influential factors that affect the walking activity of the elderly in China's megacities, thereby helping to provide optimized strategies for the healthy development of elderly city residents.

## Data Availability Statement

The datasets presented in this article are not readily available because the dataset is a part of the project “Intelligent Recognition of Street Space Quality and its Planning Application: a Case Study of Wuhan” funded by the National Natural Science Foundation of China, 2020.01-2023.12. So the dataset is confidential during this period. Requests to access the datasets should be directed to Xiaowu Lin, 973742941@qq.com.

## Ethics Statement

Ethical review and approval was not required for the study on human participants in accordance with the local legislation and institutional requirements. The patients/participants provided their written informed consent to participate in this study.

## Author Contributions

HH conceived of the study, and participated in its design and coordination. XL and TL led the manuscript preparation. XL contributed to data collection and analysis. TL and YY contributed to data collection. All authors read, contributed to, and approved the final manuscript.

## Conflict of Interest

The authors declare that the research was conducted in the absence of any commercial or financial relationships that could be construed as a potential conflict of interest.
